# Water Sorption and Structural Properties of Human Airway Mucus in Health and Muco-Obstructive Diseases

**DOI:** 10.1021/acs.biomac.3c01170

**Published:** 2024-02-09

**Authors:** Susyn J. Kelly, Vladislav Genevskiy, Sebastian Björklund, Juan F. Gonzalez-Martinez, Lara Poeschke, Maik Schröder, Georg Nilius, Stanislav Tatkov, Vitaly Kocherbitov

**Affiliations:** † Fisher & Paykel Healthcare Ltd., 15 Maurice Paykel Place, East Tamaki, Auckland NZ-2013, New Zealand; ‡ Department of Clinical Sciences, Ross University of Veterinary Medicine, Basseterre KN-0101, Saint Kitts and Nevis; § Biomedical Science, Faculty of Health and Society, Malmö University, Malmö SE-20506, Sweden; ∥ Biofilms Research Center for Biointerfaces, Faculty of Health and Society, Malmö University, Malmö SE-20506, Sweden; ⊥ Department of Applied Physics, Universidad Politécnica de Cartagena, Cartagena 30202, Spain; # Evang. Kliniken Essen-Mitte GmbH, Essen DE-45136, Germany; ¶ Universität Witten/Herdecke, Witten DE-58455, Germany

## Abstract

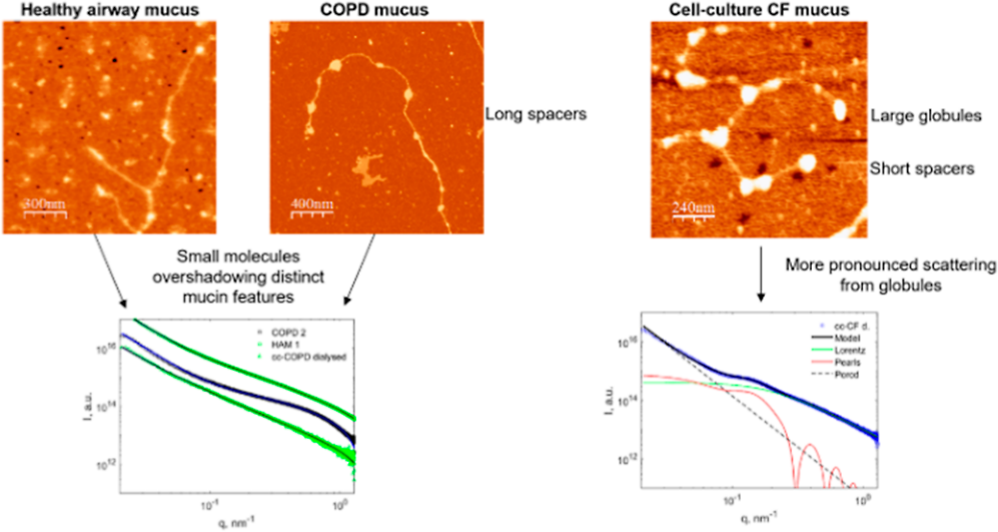

Muco-obstructive diseases change airway mucus properties, impairing mucociliary transport and increasing the likelihood of infections. To investigate the sorption properties and nanostructures of mucus in health and disease, we investigated mucus samples from patients and cell cultures (cc) from healthy, chronic obstructive pulmonary disease (COPD), and cystic fibrosis (CF) airways. Atomic force microscopy (AFM) revealed mucin monomers with typical barbell structures, where the globule to spacer volume ratio was the highest for CF mucin. Accordingly, synchrotron small-angle X-ray scattering (SAXS) revealed more pronounced scattering from CF mucin globules and suggested shorter carbohydrate side chains in CF mucin and longer side chains in COPD mucin. Quartz crystal microbalance with dissipation (QCM-D) analysis presented water sorption isotherms of the three types of human airway mucus, where, at high relative humidity, COPD mucus had the highest water content compared to cc-CF and healthy airway mucus (HAM). The higher hydration of the COPD mucus is consistent with the observation of longer side chains of the COPD mucins. At low humidity, no dehydration-induced glass transition was observed in healthy and diseased mucus, suggesting mucus remained in a rubbery state. However, in dialyzed cc-HAM, a sorption–desorption hysteresis (typically observed in the glassy state) appeared, suggesting that small molecules present in mucus suppress the glass transition.

## Introduction

Mucus is a viscous fluid that acts as a protective layer, maintaining cell hydration and preventing infection. It is regarded as a hydrogel consisting of mainly water (90–95 wt %), mucins (2–10 wt %), lipids (1–2 wt %), salts (1 wt %), small amounts of cellular debris, and other molecules.^1,2^ In the respiratory tract, the mucus covers the periciliary layer, which together forms the so-called airway surface liquid (ASL) that lines the epithelium.^3^ The low viscosity of the periciliary layer allows the cilia to beat,^4^ propelling the overlaying mucus, which entraps inhaled debris, up the airway to the larynx by so-called mucociliary transport.^3^ The mucus layer relies on effective mucus–cilia interactions for mucociliary transport and, as such, it is the mucus properties and osmotic pressure that determine how effectively the ciliary beat can move the mucus and protect the airway from infection. The mucus layer is at the air interface, and therefore, mucus properties are strongly affected by external parameters, such as relative humidity.

The primary protein components of mucus are mucins, which are continuously secreted by cells and submucosal glands in the airway epithelium.^5^ Mucins are glycoproteins that are responsible for the biophysical properties of mucus and appear to determine the function of the mucus layer. However, the structures of the mucin macromolecules are complex due to their long-range charge effects and tendency to associate. In general, mucin monomers are centered around a glycosylated peptide backbone composed of hydroxyl-rich amino acids that promote radial binding of glycans, forming a bottle-brush structure.^6^ The glycosylated peptide spacers separate two asymmetric globules, forming the barbell structure of the mucin monomer,^7^ which combine via disulfide bonding to form long multiglobule polymeric structures.^8^ Even though the structure of the mucin macromolecules influences the biophysical properties of mucus, the viscosity and elasticity of mucus are also determined by its composition, where the mucin and water content have the most influence on mucus’s gel-forming properties. The predominant gel-forming polymeric mucins found in higher concentrations in proximal airways and lower concentrations in the distal airways are MUC5B and MUC5AC,^9^ with lower amounts of MUC2.^1,10^ Taken together, mucins do not only impart biophysical properties to the ASL but also coordinate hydration between the mucus and periciliary layer.^11,12^

In muco-obstructive diseases such as chronic obstructive pulmonary disease (COPD) and cystic fibrosis (CF), mucociliary transport is compromised by the poorly hydrated ASL, caused by mucin hypersecretion and imbalanced ion transport across the epithelium.^12^ The mucin concentration in the ASL of COPD patients is at least doubled when compared to healthy and can be even greater in CF patients.^12−14^ Mucins become extensively entangled at high concentrations due to ionic fluxes in the ASL caused by the improper function of the cystic fibrosis transmembrane conductance regulator,^14^ which adjusts the volume of the ASL to maintain mucociliary function.^15^ These ionic fluxes, accompanied by increased mucin concentrations, change the biophysical properties and hydration of the ASL, resulting in ineffective mucociliary transport and mucostasis.^16,17^ The accumulated mucus blocks the bronchi, leading to hypoxia in the epithelial cells, which could be central to the pathogenesis of persistent mucus accumulation in muco-obstructive lung diseases.^18^ The mucostasis and subsequent exacerbations are one of the strongest predictors of mortality in advanced COPD patients.^19−21^ A recent observational study concluded that mucus plugs that completely occlude medium- and large-sized airways in COPD patients are associated with high all-cause mortality.^22^ A better understanding of mucus properties in health and disease will provide insight into the physiology of mucociliary transport.

Mucus has been difficult to study with common physicochemical methods due to limited sample volumes, especially for healthy airway samples in the absence of hypersecretion, and the size of mucins and their propensity to aggregate, where they form mucoadhesive interactions with other molecules. To date, research on mucus has focused on the characteristics of isolated/purified mucins.^6,23−25^ Quartz crystal microbalance with dissipation (QCM-D) analysis has been used to investigate the water sorption properties of mucin films,^24,25^ in environmental conditions similar to those found in the airway during normal breathing.^26,27^ The water sorption isotherms of mucins have been determined by humidity scanning (HS) QCM-D and sorption calorimetry, which have provided insights into the factors that control water uptake^23,24^ and showed that the onset of sorption–desorption hysteresis coincides with the hydration-induced glass transition.^25^

Synchrotron small-angle X-ray scattering (SAXS) and small-angle neutron scattering (SANS) have proven to be useful techniques in studying protein structures in solution to better understand how these structures can impart physical properties in biological systems.^28^ Fitting models to the scattering curves from mucins in solution identified an approximate geometric structure. Griffiths and co-workers^29^ presented a so-called sphere-power law model that describes hydrophobic dispersed spheres and glycosylated chain structures using various concentrations of pig gastric mucin in solution. Later, Znamenskaya et al.^6^ presented another model that emphasizes the contribution of the scattering from the carbohydrate side chains. Complementary to SAXS analysis is atomic force microscopy (AFM) to image and measure nanostructures. AFM has been used to characterize mucins from human tracheobronchial epithelium cell cultures,^30,31^ isolated pig gastric mucin, and bovine submaxillary gland mucins.^8^ An improved understanding of the sorption properties and mucin structures found in mucus from muco-obstructive diseases that impair mucociliary function may help advise more targeted treatment strategies that adjust the mucus-water composition, thereby promoting effective mucus–cilia interactions for mucociliary transport.

The present work tested the hypothesis that the water sorption properties and nanostructures of human airway mucus in muco-obstructive diseases may vary from healthy airway mucus (HAM). The study was performed on available mucus samples taken from patients with muco-obstructive disease (COPD), patients without lung disease following elective surgery (healthy), as well as from in vitro grown human bronchial epithelium (healthy, COPD, and CF) using QCM-D, SAXS, and AFM.

## Materials and Methods

### Samples

Mucus samples were collected from patients at Kliniken Essen-Mitte, Essen, Germany, and from reconstructed airway epitheliums grown in vitro (MucilAir, Epithelix, Switzerland). The patient samples were collected as part of a registered trial (clinicalTrials.gov, NCT04703023) approved by the Ethics Committee of University Witten/Herdecke (Nr 16/2020). Informed consent was obtained from all patients. The healthy airways mucus (HAM 1 and HAM 2) samples were collected from a healthy patient undergoing elective surgery (sample volume <0.5 mL), using the mucus that had accumulated on the endotracheal tube, as described in Markovetz et al.^32^ and briefly here; soon after the endotracheal tube was removed from the patient, the end of the tube was cut off (about 10 cm in length), placed in a large conical tube, and then quickly sealed to prevent dehydration. The sample was then placed in a centrifuge with a centrifugal force of 4000*g* for 5 min. A 1000 μL pipet was used to draw up the mucus that had accumulated in the conical tube to transfer the sample into a 1.5 mL Eppendorf tube. The Eppendorf tube was then rapidly cooled to prevent conformational changes^33^ and stored at −80 °C. The COPD mucus samples were collected from a patient’s exacerbation visit (COPD 1, sample volume >0.5 mL) and from a control visit for a planned bronchoscopy (COPD 2, sample volume >0.5 mL). Samples were collected with a bronchoscope with a wide suctioning channel and placed in Eppendorf tubes, which were also submerged in dry ice and stored at −80 °C.

The MucilAir mucus samples from the reconstituted human airway epithelium cell cultures (cc) (sample volume 1 mL) were prepared from epithelial cells isolated from pieces of whole lung tissue (post-mortem, collected by Epithelix partner centers with consent from the donor or next of kin) and purified using enzymatic digestion. These primary cells were then seeded on polyester membrane cc inserts (Corning Transwell, CLS3470, Merck, USA) and submerged until confluence with Epithelix hAEC media (EP09MM, serum-free, Epithelix, Switzerland) for 1 week. The cc were then switched to an air liquid interface and cultured in MucilAir Media (EP05MM, serum-free, Epithelix, Switzerland) until full differentiation was achieved after 5 weeks. The mucus samples were collected by lavaging the mucosal surface of the cc from healthy (cc-HAM), COPD (cc-COPD), and CF (cc-CF) airways with 55 μL of isotonic saline solution on the apical surface. Samples denoted as cc-HAM, cc-COPD, and cc-CF were stored at −80 °C.

For dialysis, 0.5 mL of the cc-HAM, cc-COPD, and cc-CF samples was diluted in 1.5 mL of Milli-Q water before being loaded into the filter unit with a 3 kDa molecular weight cutoff (Amicon Ultra-15, Sigma-Aldrich, Germany); 15 mL of Milli-Q water was then added on top of the filter unit before centrifugation at 4500*g* for 30 min. The filtered solution was removed, and the abovementioned procedure was repeated three times. The purified samples were then collected in 2 mL Eppendorf tubes and stored at −80 °C.

### Quartz Crystal Microbalance with Dissipation Monitoring

#### QCM-D Analysis

QCM-D experiments were performed with a Q-Sense E4 instrument equipped with a humidity module (QF401, Biolin Scientific AB, Sweden) to measure the water sorption isotherms at room temperature (25 °C). This technique is described in detail elsewhere^34,35^ and has previously been employed by us and others to investigate the hydration of thin films of various (bio)materials, such as lyophilized mucins,^24,25^ lysozyme,^25,36^ mesoporous silica,^37^ lipids/surfactants,^38,39^ and latex coatings.^40^ The silicon dioxide 5 MHz quartz crystal sensor (QSX303, Biolin Scientific AB, Sweden) was used for all measurements. QCM-D analysis works by applying an oscillating force to the quartz crystal sensor, where the change in the resonance quartz crystal frequency (Δ*f*) is linearly proportional to the change in mass of the film, assuming the film behaves as a solid (the Sauerbrey equation)
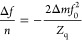
1In eq 1, Δ*f*/*n* is the frequency change normalized to the overtone number, *f*_0_ is the fundamental frequency of the quartz crystal (approximately 5 MHz for the sensors used here), *m* is the areal film mass (kg·m^–2^), and *Z*_q_ is the acoustic impedance of quartz (8.8 × 10^6^ kg·m^–2^).

To create the conditions for water sorption and desorption from the mucus film, solutions with controlled concentrations of LiCl (Lot: MKCG0360, Sigma-Aldrich, Germany) were injected in sequence to either increase or decrease the water activity. The dilutions were made based on experimental data on water activity as a function of LiCl concentration,^41^ which were fitted with a polynomial function, as previously described.^36^ The LiCl solutions used in this experiment had the following water activities (*a*_w_): 0.11, 0.25, 0.40, 0.55, 0.71, 0.84, 0.94, 0.98, and 0.99, where *a*_w_ is related to the relative humidity (%) simply by multiplying by 100. Each of the abovementioned solutions was injected with a peristaltic pump (Ismatec IPC4, Fisher Scientific, UK) at a flow of 0.1 mL/min until the overtones (*n* = 1, 3, 5, and 7) had stabilized. The starting and ending relative humidity values (0%) were obtained by introducing dry N_2_ into the chamber.

#### Humidity Scanning QCM-D

A detailed description of the HS QCM-D method for water sorption–desorption isotherms can be found elsewhere.^35^ In brief, the method works in a manner similar to that described above for the stepwise injection of aqueous LiCl solutions with specified water activities (i.e., specified LiCl concentrations). In HS mode, however, the aqueous LiCl solution is continuously diluted (sorption mode) or concentrated (desorption mode), after which the solution is introduced into the humidity module via tubes connected to a peristaltic pump to control the desired humidity.

#### Sample Preparation

Mucus films were deposited onto the quartz crystal sensor by spin coating with an aqueous solution of the sample to obtain appropriate film thicknesses.^25^ To achieve appropriately thin films, the sample concentration was lowered by diluting all the samples 1:15 using Milli-Q water. An exception was made for HAM 1 sample, collected after endotracheal intubation, which was diluted 1:30 times due to a higher number of insoluble solids. All samples were spin coated onto the quartz crystal sensor by applying 20 μL of the aqueous samples, repeated five times, while the sensor was spun at 1200 rpm (a spin coater developed in-house). The coated quartz crystal sensor was then left overnight in a silica gel desiccator to allow the solvents to evaporate, resulting in dry mucus films of 20–80 nm thicknesses.

#### Analysis

Prior to mucus sorption measurements, uncoated QCM-D sensors were used to establish baseline measurements. Next, the sensors were unmounted to allow for spin-coating with mucus films. The mucus-coated sensors were then remounted into the instrument and measured under N_2_ flow (0% relative humidity) to determine the dry mass of the coated layer. The dry mass was used to determine the water content of the mucus film due to water adsorption when exposed to different relative humidities generated by the LiCl solutions with different water activities, as described above. The mucus water content upon exposure to different humid environments was expressed as a mass fraction of the absorbed water mass over the total mass of the layer
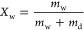
2In eq 2, *m*_w_ is the mass of the adsorbed water and *m*_d_ is the mass of the dry sample on the quartz crystal sensor. Since the mass is correlated with the change in frequency, the equation above can be rewritten in terms of frequency changes
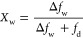
3where Δ*f*_w_ is the frequency change caused by the water adsorption and *f*_d_ represents the frequency shift caused by the dry layer of mucus; i.e., *f*_d_ = *f*_s_ – *f*_e_, where *f*_s_ is the frequency corresponding to the coated sensor in dry conditions (0% relative humidity) and *f*_e_ is the frequency from the uncoated sensor. Given the high mass sensitivity of the instrument (17.7 ng/cm^2^ Hz), the uncertainty related to the adsorbed water content was calculated by assuming the mass-uptake measurement was unaffected by errors. As such, there was no error associated with the frequency change caused by water sorption. The error propagation taken into consideration was ±20 Hz, which was observed in the results only during the installation and removal of the QCM-D sensor during the baseline measurements (*f*_d_). The error affecting *f*_d_ can therefore be calculated as a root of the sum of squares of the two uncertainties (during the installation of the empty sensor for the baseline measurement and during the installation of the same coated sensor for the sorption measurement)
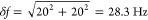
4The uncertainty of the water content was calculated as the derivative of *X*_w_ with respect to *f*_d_
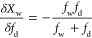
5

#### Atomic Force Microscopy

AFM images of the mucus samples were collected using multimode scanning probe microscopy with a Nanoscope V, MultiMode 8 (Bruker), and silicon cantilevers with a nominal resonance frequency of 300 kHz (RTESPA-300, Bruker, USA). Tapping mode with a cantilever force constant of 30 N/m (calibrated using the Sader method) and oscillation amplitude of approximately 14–40 nm (some images required higher oscillation amplitudes) was used to image the mucus samples with a scan rate of 1 Hz. The images show the topography channel; amplitude and phase are not shown as they did not provide relevant information. All images were obtained at room temperature.

##### Sample Preparation

Mucus samples were diluted with distilled water to a concentration of 10^–5^ wt %. The diluted mucus samples were then drop-coated on a mica surface and left to dry in a desiccator overnight. Due to the limited sample volumes, we were not able to perform pH measurements of the samples to inform adjustments to the mica surface properties, which have been shown to improve sample adherence.^8^

##### Analysis

AFM topography images were analyzed with WSxM software^42^ to determine the volume of the imaged mucin molecules. The volume of the mucin molecules was calculated using measurements of the equivalent globule radius (*r*_globule_), the equivalent spacer radius (*r*_spacer_), and length (*l*_spacer_), which were used to calculate the globule volume (*V*_g_) and the spacer volume (*V*_S_) according to the following equations, where the equivalent globule volume was assumed to be a sphere, and the spacer volume was assumed to be a cylinder
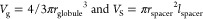
6

### Small-Angle X-ray scattering

Small-angle X-ray scattering (SAXS) was performed at the I22 beamline (Diamond Light Source, UK, Proposal SM23182) using a Pilatus P3–2 M detector. The sample to detector distance was set to 6 m for a *q*-range of 0.02–1.3 nm^–1^, where *q* = (4π/λ) sin (θ/2) and θ is the scatter angle and λ is the X-ray wavelength (12.4 keV). Samples were loaded into polycarbonate capillaries with a 2 mm internal diameter (Precision Extrusion Inc., USA) mounted onto guide rails, sealing the capillary ends, and forming a ladder arrangement for SAXS measurements. Measurements were performed on each sample in triplicate with a 1 mm spacing along the capillary. All measurements of the transmitted intensities were performed at room temperature. The two-dimensional scattering patterns were normalized with background subtraction using a blank capillary and a water standard and azimuthally averaged for scattering intensities as a function of the scattering vector (*I*(*q*)). Scattering curves were presented as double logarithmic plots.

### Sample Preparation

All of the samples were thawed at room temperature. HAM 1, HAM 2, COPD 1, and COPD 2 mucus were divided into two groups: one group contained original/native samples for SAXS measurements, and the other group contained diluted samples (1:10 with Milli-Q water), which were centrifugally separated for SAXS measurements of the supernatant (containing the water-soluble mucins) and pellet (other water-insoluble mucins and debris such as DNA and cells). The dilution was performed with approximately 50 mg of mucus, diluted into 450 μL of Milli-Q water, and vortexed for about 1 min to homogenize the solution. Samples were filled into the capillaries with a needle and syringe and momentarily centrifuged to remove any air bubbles. Due to limited sample volumes, centrifuged samples from only HAM 1 and COPD 1 were possible. The cc-HAM, cc-COPD, and cc-CF samples were investigated in their original, centrifuged, and dialyzed states (as described above) to remove the additional salts that were introduced when the reconstituted human upper airway epithelium was lavaged during collection. The dialyzed samples were thawed and loaded into capillaries at the synchrotron.

### Analysis and Modeling of SAXS Data

SasView (version 5.0.4) was used for fitting the SAXS data. The model used to fit the mucus scattering data should include contributions from two predominant structures: a glycosylated peptide spacer and protein globules. This was similar to the approach used by Griffiths et al.^29^ and agreed with the structures observed in AFM images
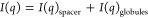
7Here (unlike the work by Griffiths et al.^29^), the scattering contribution from the glycosylated spacer (*I*(*q*)_spacer_) was expressed using the correlation length model,^43^ consisting of two contributions: the Porod term and the Lorentzian term
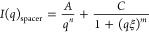
8where *q* is the scattering vector *n* and *m* are the Porod and Lorentzian exponents, respectively. In the framework of this model, we suggest that the first term describes the overall shape and aggregation of the mucin molecules (with a possible contribution from other large-size aggregates). The second term describes scattering from mucin side chains and scattering due to polypeptide backbone–side chains interactions.

The scattering contribution from the mucin globules (*I*(*q*)_globule_) was described using the linear pearls model,^44^ which reduces to the following expression when considering only two connected globules
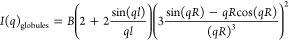
9where *R* is the radius of the sphere and *l* is the distance between the edges of the globules. Thus, the concentration-dependent structure factor was excluded from the model, and a fixed distance between globules defined by the spacer size was assumed.

The mass fractal dimensions *n* and *m* can be used to determine the Flory exponents *v* for different length scales
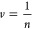
10In general, when the polymer chain straightens and obtains conformations similar to those of rods, the Flory exponent is close to 1.0. Poor polymer–solvent interactions will cause the polymer to compact and for a globular conformation, and the Flory exponent will be closer to 1/3. If the polymer conformation is in a random coil arrangement, the Flory exponent will be around 1/2; self-avoiding walk conformations correspond to a Flory exponent of 0.588 and hence a *n* value of 1.70.

## Results and Discussion

### Water Sorption Isotherms of Airway Mucus

#### Sorption Isotherms of Different Types of Mucus and Mucins

Here, we present the first water sorption isotherms of human mucus collected during endotracheal intubation (HAM) and bronchoscopy (COPD 1 and COPD 2). Mucus samples from cc-HAM, cc-COPD, and cc-CF were examined.

**Figure 1:**
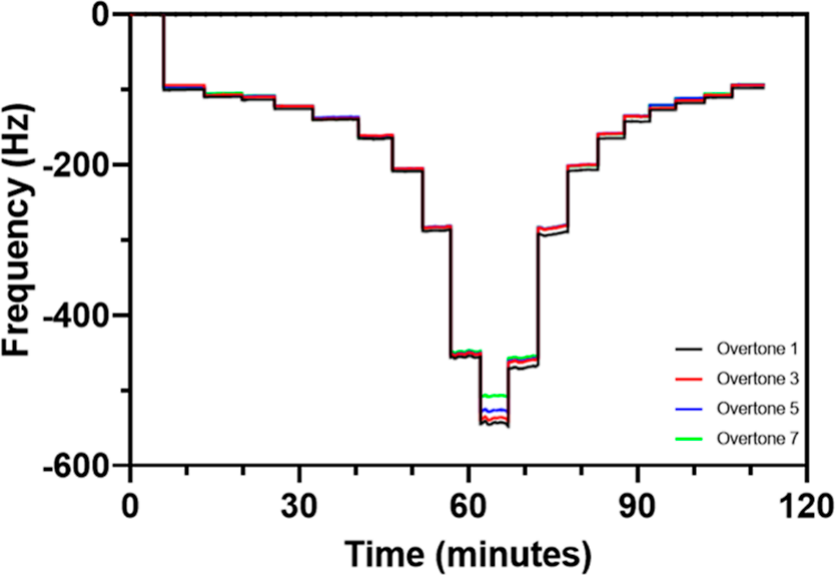
Representative results from water sorption–desorption measurements on mucus films using a QCM-D. The initial decrease of the frequency (normalized for overtones) occurs due to water uptake from the surrounding vapor phase, where the relative humidity is adjusted in several steps between 0 and 99% (sorption mode). Subsequent stepwise increase of the frequency corresponds to a 99–0% decrease in relative humidity (desorption mode). Data from an approximately 15 nm thick mucus film obtained from cc-HAM.

The QCM-D sorption isotherm experiments performed in this study investigate the water contents in different mucus films under isothermal conditions (25 °C) at relative humidities between 11 and 99% set by controlled dilutions/concentrations of a LiCl solution (see an example in Figure 1). The mucus films deposited onto the quartz crystal sensors, used to determine the sorption–desorption isotherms, were thinner compared to those reported in previous studies^45^ (20–80 nm rather than the 200–1000 nm). Measurements using thin films can be affected by small alterations of the resonance frequency caused by mounting the sensor into the QCM-D module, introducing errors in estimating the dry mass of the film. The error has been approximated as a change of around 3 nm in the film thickness. On the other hand, thick mucus films can exhibit more complex viscoelastic behavior, without clear overtones and unstable resonance frequencies, which become more unstable at higher levels of hydration. Thin films allow the mucins to distribute more evenly across the film, with better contact between the film and the crystal sensor surface, thereby meeting the requirements of the Sauerbrey equation.^46^

**Figure 2:**
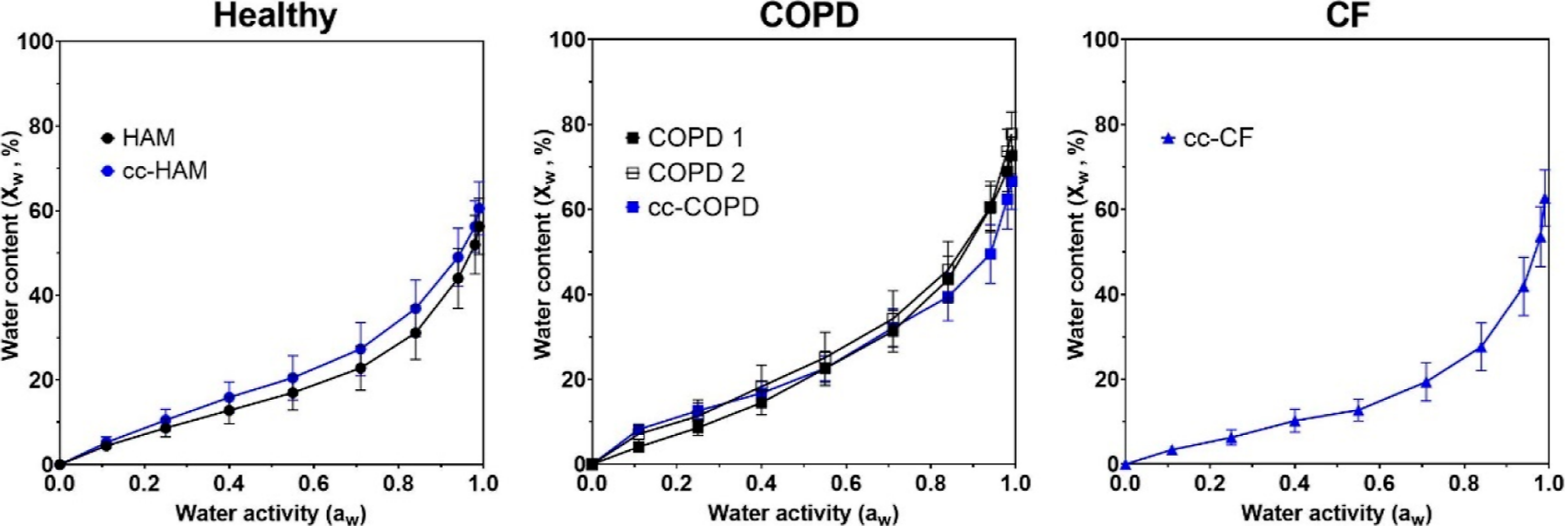
Average water sorption isotherms at 25 °C of mucus films (20–80 nm) from HAM 1 (black) and cc HAM (blue), COPD mucus collected during bronchoscopy (COPD 1, filled black square and COPD 2, black square), cc-COPD (blue square), and cc-CFCF mucus. Due to the absence of hysteresis, only the sorption is reported. Error bars correspond to standard deviations calculated from several measurements for every step of water activity obtained during the 10 min once stable readings were reached.

The sorption properties of the HAM, COPD 1, and COPD 2 mucus, and the cc-HAM, cc-COPD, and cc-CF mucus were in good agreement within a sample type, and there were significant differences between the COPD, CF, and HAM sample types (Figure 2 and Table S1). There were no significant differences neither between the sorption properties of the HAM and cc-HAM nor COPD 1, COPD 2, and cc-COPD (*P* < 0.05) (Supporting Information, Table S1). Further, COPD 1 and COPD 2 were not significantly different from one another (*P* > 0.05) (Figure 2B). The COPD samples were significantly different from the HAM samples (*P* > 0.05) and, similarly, the cc-COPD and cc-CF were significantly different from the cc-HAM (*P* > 0.05). These significant differences between the sample types suggest different sorption characteristics of mucus from COPD and CF airways. The maximum water content of the mucus samples when exposed to 99% relative humidity (*a*_w_ = 0.99) was highest in COPD 1 and COPD 2 samples (73 and 78 wt %, respectively), while the maximum water content for the HAM sample was lower (56.3 wt %). Similarly, for the cc samples, the cc-COPD mucus had a higher water content than the cc-HAM (67 wt % vs 63 wt %, respectively), while the cc-CF mucus had the lowest water content (61 wt %) when exposed to 99% relative humidity.

#### Sorption–Desorption Hysteresis

The sorption–desorption characteristics of the different mucus types were used to determine whether mucus samples exhibit sorption/desorption hysteresis caused by isothermal hydration-induced glass transition. Previously, the isothermal hydration-induced glass transition composition (when a polymer transitions from a glassy to rubbery state) has been found to be in good agreement with the onset of hydration-induced sorption–desorption hysteresis.^25^ Contrary to previous sorption–desorption isotherms of mucins, which showed hysteresis,^25^ all mucus samples (HAM, COPD 1, and COPD 2) and cc samples (cc-HAM, cc-COPD, and cc-CF) presented here showed no hysteresis (Figure 2). This implies that no hydration-induced glass transition at the temperature and humidity used in these experiments was observed and that the mucus remained in a rubbery state. The absence of a glass transition is an important physiological property of mucus when acting as a protective layer for the airway epithelium.

Mucus acts as a water reservoir in ASL. The addition or removal of water can change the viscoelastic properties of the ASL and thus influence the efficiency of the ciliary action as well as the mucus–cilia interactions for mucociliary transport.^47−50^ The lack of hysteresis and isothermal hydration-induced glass transition is of physiological importance since it highlights the protective function that mucus properties impart on the airway surface by maintaining a rubbery state, even when exposed to low relative humidity environments where mucociliary clearance is compromised.^51^ By maintaining a rubbery state, the overlaying mucus protects the airway epithelium from desiccation, which, with repeated hyperpnea with cold dry air, has been shown to result in airway remodeling, similar to that seen in asthma.^52^ If the airway is exposed to low relative humidity environments for a short period of time and then quickly restored to physiological conditions, mucus properties are restored and mucociliary function resumes, all without lasting effects of cellular damage on the airway epithelium.^51^

**Figure 3:**
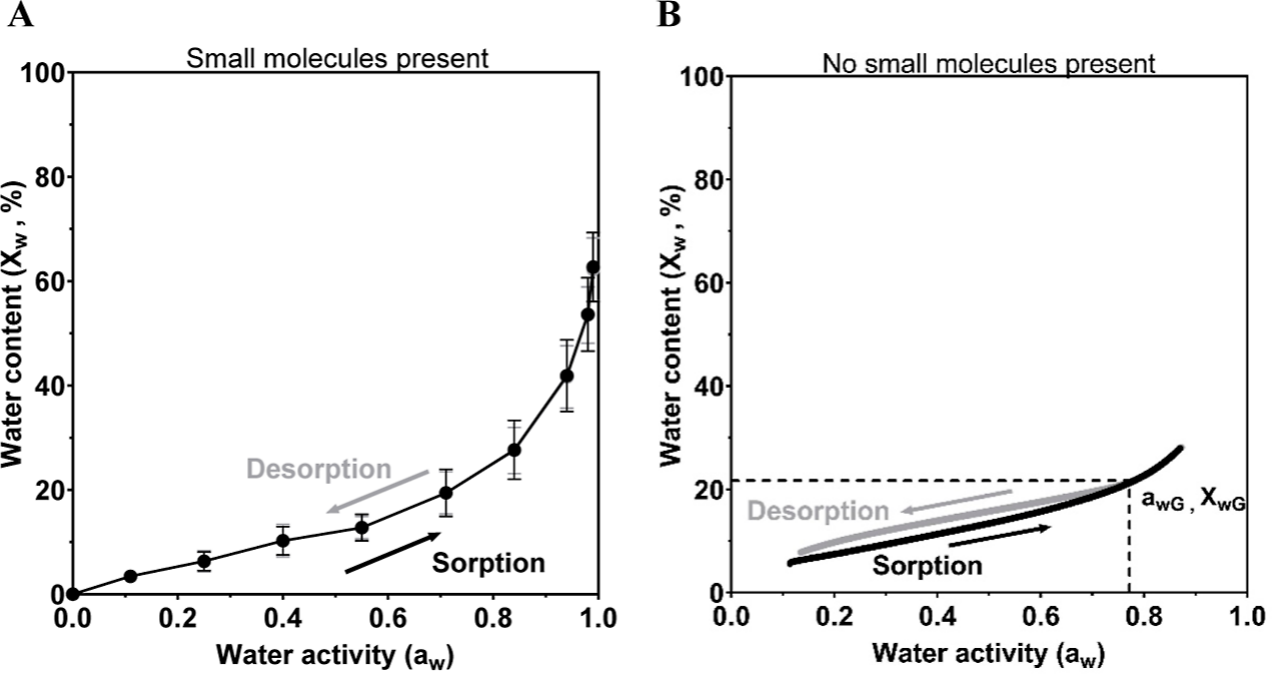
Sorption (black) desorption (gray) isotherm (25 °C) from the (A) undialyzed/native cc-HAM, film thickness 35 nm, showing no hydration-induced sorption–desorption hysteresis (desorption curve is overlaid by the sorption curve) or glass transition point using stepwise LiCl dilutions, and (B) the dialyzed cc-HAM, film thickness 285 nm, showing hydration-induced sorption–desorption hysteresis and a glass transition point (*a*_wG_, *X*_wG_), using continuous data from continuous LiCl dilutions.

In this study, we performed further experiments to better understand why no hydration-induced hysteresis was observed in neither the cc samples nor the mucus samples obtained from patients. Dialysis was applied to the cc-HAM samples, effectively removing small molecules such as salts and carbohydrates from the mucus. Then water sorption measurements on dialyzed samples were studied using QCM-D to compare with the sorption isotherms of native samples (Figure 3). A clear hysteresis appears when samples have been dialyzed (Figure 3B), where the water content during sorption is lower than the water content during desorption. The onset of hysteresis is related to the isothermal hydration-induced glass transition and appears at 25 °C and 77% relative humidity, corresponding to a water content of approximately 20 wt % in the cc-HAM sample (Figure 3B). Hysteresis was not present in samples that were not dialyzed (Figures 2 and 3A), suggesting that small molecules in the mucus suppress sorption–desorption hysteresis.

Mucus containing small molecules can maintain an intact mucus barrier with consistent sorption and desorption properties at varying humidities. The water activity at the onset of hysteresis in the dialyzed cc-HAM sample (Figure 3A) is in good agreement with data by Björklund and Kocherbitov,^25^ who also reported hysteresis and a similar glass transition of two purified mucin types (PGM and BSM), and Znamenskaya and co-workers^23^ who reported hysteresis in QCM-D films and bulk samples.

The rationale for the hysteresis seen here could be explained by the sample’s inability to reach equilibrium state in the glassy state. The small molecules (including salts, small sugars, etc.) not only directly affect sorption isotherms by binding a certain amount of water but also act as plasticizers in mucus, shifting the glass transition to lower temperatures and water contents. Hence, in the presence of small molecules, the mucus remains in an equilibrium rubbery state, and hysteresis is not observed. However, when these small molecules are removed from the mucus by dialysis, the glass transition occurs at a higher water content, and in the glassy state, the absorbed amount depends on the sample’s hydration history. In summary, not only mucins that are important for mucus properties and its ability to protect the airway but also the small molecules that are present in the mucus appear to increase water sorption capacity and water holding ability.

#### AFM

**Figure 4:**
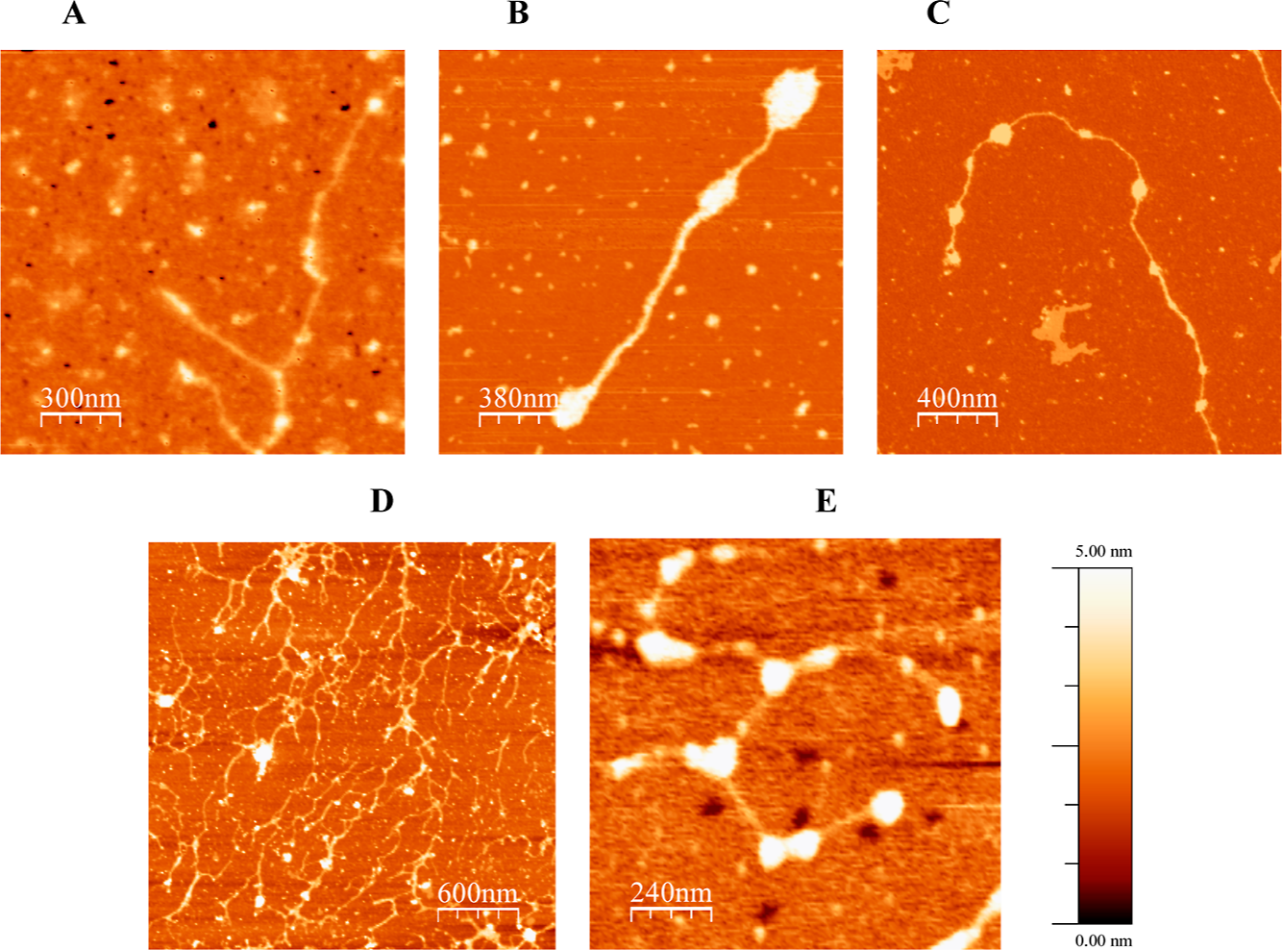
Representative AFM topography images of mucins deposited onto a mica surface from mucus collected: (A) HAM, (B) cc-HAM, (C) COPD 1, (D) cc-COPD, and (E) cc-CF. Mucus was diluted 10^–5^ wt % and deposited onto a mica surface for all images.

AFM was used to visualize structural differences and measure dry molecule volumes of mucins found in mucus collected after endotracheal intubation (HAM) or during bronchoscopy (COPD) and the mucus grown on cc-HAM, cc-COPD, and cc-CF. The AFM topography images of the mucins, deposited and dried onto a mica surface, revealed typical globular structures linked by spacers, or so-called barbell structures, organized into fiber-like structures in all mucus samples (Figure 4). The volume of the dry mucin molecules on the mica surface was determined, allowing the globule radius and spacer length to be estimated (Table 1). In addition to the prominent mucin structures in the images, small molecules (approximate radius 1–6 nm) were also present in these samples, seen as spheres in the background (Figures 4 and S1), an observation that was not made in the background of AFM images of purified PGM and BSM.^7^

**Table 1:** Summary of the Equivalent Globule Radius (*r*_globule_), Spacer Radius (*r*_spacer_), and Spacer Length (*l*_spacer_) of Mucin Molecules Observed with Atomic Force Microscopy Topography Images (the Presented Error Values Are Standard Deviations)

sample	collection method	*l*_spacer_ [nm]	*r*_spacer_ [nm]	*r*_globule_ [nm]^a^	*V*_g_/*V*_S_^b^
HAM	after endotracheal intubation	260 ± 94	3.3 ± 0.6	10.8 ± 0.6	0.6
cc-HAM	reconstituted human airway epithelium	444 ± 125	5.5 ± 0.6	20.5 ± 4.7	0.9
COPD 1	COPD exacerbation bronchoscopy	339 ± 97	3.5 ± 0.6	9.2 ± 3.7	0.3
COPD 2	planned bronchoscopy	300–500	3–4	8–15	0.4
cc-COPD	reconstituted human airway epithelium	146 ± 49	2.5 ± 0.3	12.7 ± 2.7	3.0
cc-CF	reconstituted human airway epithelium	144 ± 42	2.8 ± 0.2	17.3 ± 0.7	6.0

a Radius of a small globule. Radius of larger globules is thought to be caused by spacers wrapping around the globules.

b The ratio between the average globule (*V*_g_) and the average spacer (*V*_S_) observed in the sample.

The AFM topography images (Figure 4) and mucin molecule measurements presented here (Table 1) are comparable to other reports in the literature,^53^ where the mucin globule radius across all sample types was typically around 10 nm. While the globule radius remained similar across the samples, the organization, spacer length, and total length varied, especially in the cc-CF. The general structure of the HAM and COPD mucins and the cc-HAM and cc-COPD mucins appeared similar (Figure 4A–D) with globules and spacers in a mostly linear, fiber-like structure. The mucins observed in the cc-HAM appeared to have more variability in globule sizes compared to the HAM but had similar total lengths (approximately 2000 and 1600 nm, respectively). The mucin molecules in COPD 1 and COPD 2, however, had longer total lengths (approximately 3300 nm) with multiple globules linked together by spacers (Figure 4E). It should be noted that the total length of the mucins could be longer or shorter than those mentioned above, as the chains extend beyond the field of view in some images, and/or the sample preparation (dilution) may have resulted in chain fragments. The overall organization of the cc-CF mucin molecules was markedly different, appearing more condensed with a less elongated fiber-like structure. The cc-CF globule radius (12–18 nm) was slightly higher than those found in the HAM (8–10 nm) and COPD 1 and COPD 2 (10 nm) samples, moreover, the notably shorter spacers (120–200 nm vs 120–500 nm in the HAM and COPD mucus) could contribute to the overall structure appearing condensed and lacking a prevailing linear organization like that observed in HAM and COPD mucins.

### Small-Angle X-ray Scattering Results

#### Overview of the Results

**Figure 5:**
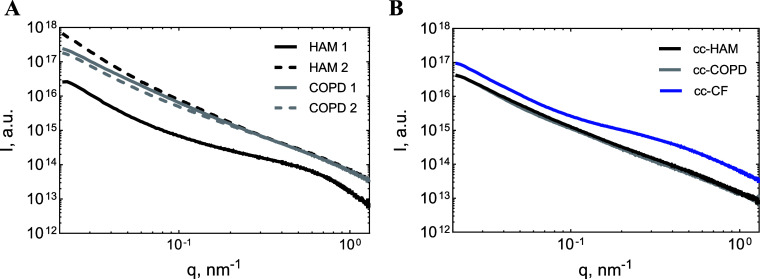
Selected original mucus scattering profiles over the measured *q*-range (0.01–1.4 nm^–1^) of (A) HAM 1 (black), HAM 2 (black dashes), COPD 1 (gray), and COPD 2 (gray dashes) and (B) cc-HAM (black), cc-COPD (gray), and cc-CF (blue). Full sample datasets are listed in Figure S2.

SAXS profiles (Figure 5) from all original patient and cc mucus samples had relatively consistent slopes at low *q* and a broad bump at high *q*. For most samples, the low-*q* slope in log–log coordinates was in the range of 2.2–2.8, which corresponds to Flory exponents of 0.36–0.45 (more details in Table S2). Since branched polymer systems have expected Flory exponent values in the range of 0.4–0.45,^54^ this part of the SAXS curves corresponds to scattering from branched networks of mucin molecules (compare with Figure 4). Several samples, for example, HAM 2, showed a steeper slope (ranging between 3.2 and 3.8), probably due to impurities, aggregates, and cellular debris present in patient samples. The broad bump in the region between 0.2 and 1.0 nm^–1^ was present in all samples and more prominent in HAM 1 and cc-CF.

**Figure 6:**
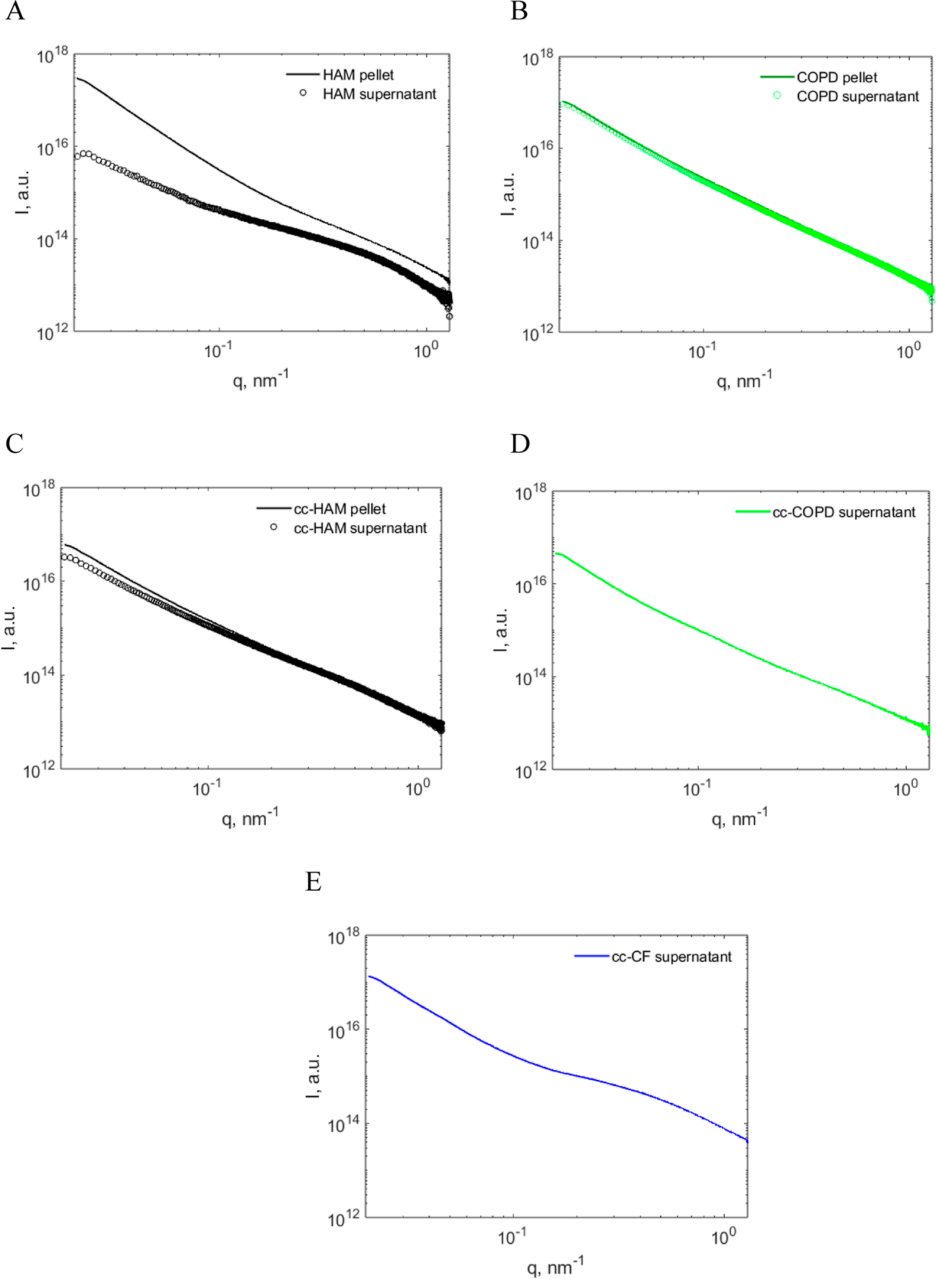
Representative supernatant and pellet centrifuged mucus scattering profiles of (A) HAM 1, (B) COPD 1, and cc samples from (C) cc-HAM, (D) cc-COPD, and (E) cc-CF.

Small molecules in the native mucus samples appear to have a significant influence on mucus properties (reported above and in the QCM-D and AFM analyses) and appear to overshadow any distinct mucin structures in SAXS analysis (Figure 5). These small molecules are likely to be other components of mucus, such as salts, small/short carbohydrates, and lipids.^55^ The small molecules also play an important role in mucus binding and may affect its properties.^56^ To better analyze the mucin structures in the various mucus samples and evaluate the effect of small molecules, we used two separation/purification methods: centrifugation and dialysis. First, centrifugation was used as a separation/purification step to partition the soluble and insoluble components of mucus into the supernatant and the pellet, respectively. A comparison of SAXS patterns obtained from the supernatant and pellet is presented in Figure 6.

The scattering profile from the patient and cc mucus pellet typically had greater intensities than the corresponding supernatants (Figure 6), which can be explained by a higher total content of biomolecules compared to the supernatant. This difference was larger for patient samples since they contain more impurities that can partition between the supernatant and pellet. Moreover, the pellet samples had a higher slope at low *q* values, which can be attributed to large aggregates or cell debris. Furthermore, in the supernatant, a more well-defined broad peak or bump at high *q* (0.6 nm^–1^) is visible in both the HAM and cc-HAM (Figure 6A,C) compared to the original sample scattering profiles in Figure 5. A less pronounced bump at high *q* also appears in the COPD 1 mucus and cc-COPD mucus supernatant (Figure 6B,D). No data was collected from the cc-COPD and cc-CF pellets since they were too solid to be pipetted into the capillaries used for SAXS analysis.

**Figure 7:**
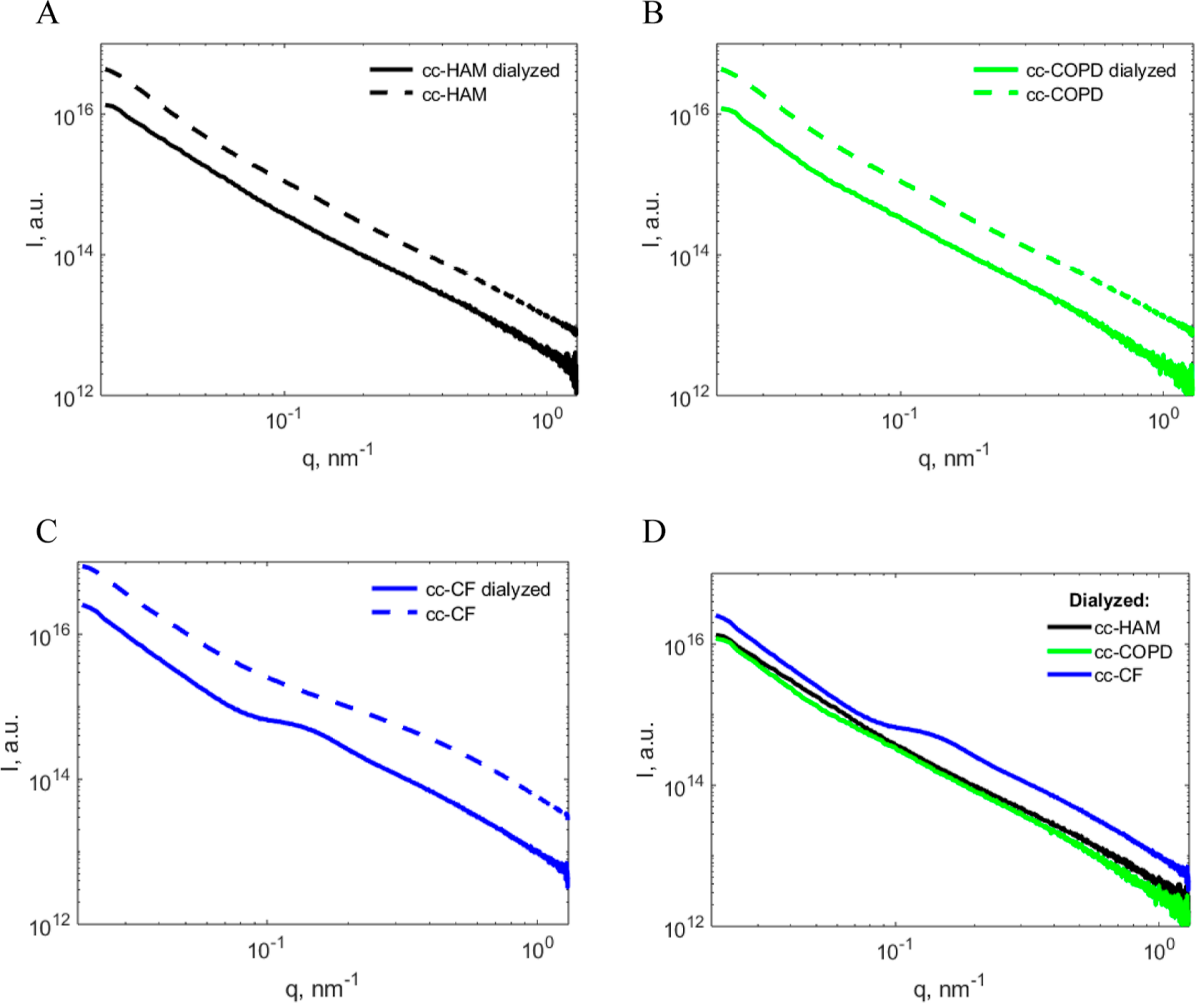
(A–C) Representative cc mucus scattering curves of the dialyzed samples (solid lines) in comparison with the original nondialyzed samples (dashed lines). (D) Comparison of the three types of dialyzed samples. In each curve, the intensity is an average of four experiments. Full sample datasets are available in Figure S4.

While centrifugation can remove suspended solids (and possibly partition macromolecules according to their sizes) in the mucus, the removal of salts, which are known to influence mucin structures,^57^ and other small molecules requires dialysis. Due to limited sample, the volumes of HAM 1, HAM 2, COPD 1, and COPD 2 mucus from patients, no further experiments were possible with these sample groups. Thus, only the cc-HAM, cc-COPD, and cc-CF mucus samples were dialyzed and subjected to further SAXS analysis (Figure 7).

This data shows that dialysis causes a similar decrease of scattering intensity for all three types of samples (cc-HAM, cc-COPD, and cc-CF). This can be explained by two factors: first, a decrease of mucin concentration from dilutions performed during the dialysis procedure; and second, the removal of small molecules and oligomers (the molecular weight cutoff of the membrane was 3 kDa). Dialysis of cc-HAM and cc-COPD mostly shifted the intensity downward, while dialysis of cc-CF resulted in an additional bump between 0.1 and 0.2 nm^–1^. Since our AFM data suggested that CF samples feature much more pronounced contributions from globules, we hypothesize that the bump in the SAXS data has the same origin. We discuss it more quantitatively in the following section.

#### Modeling of the SAXS Data

**Figure 8:**
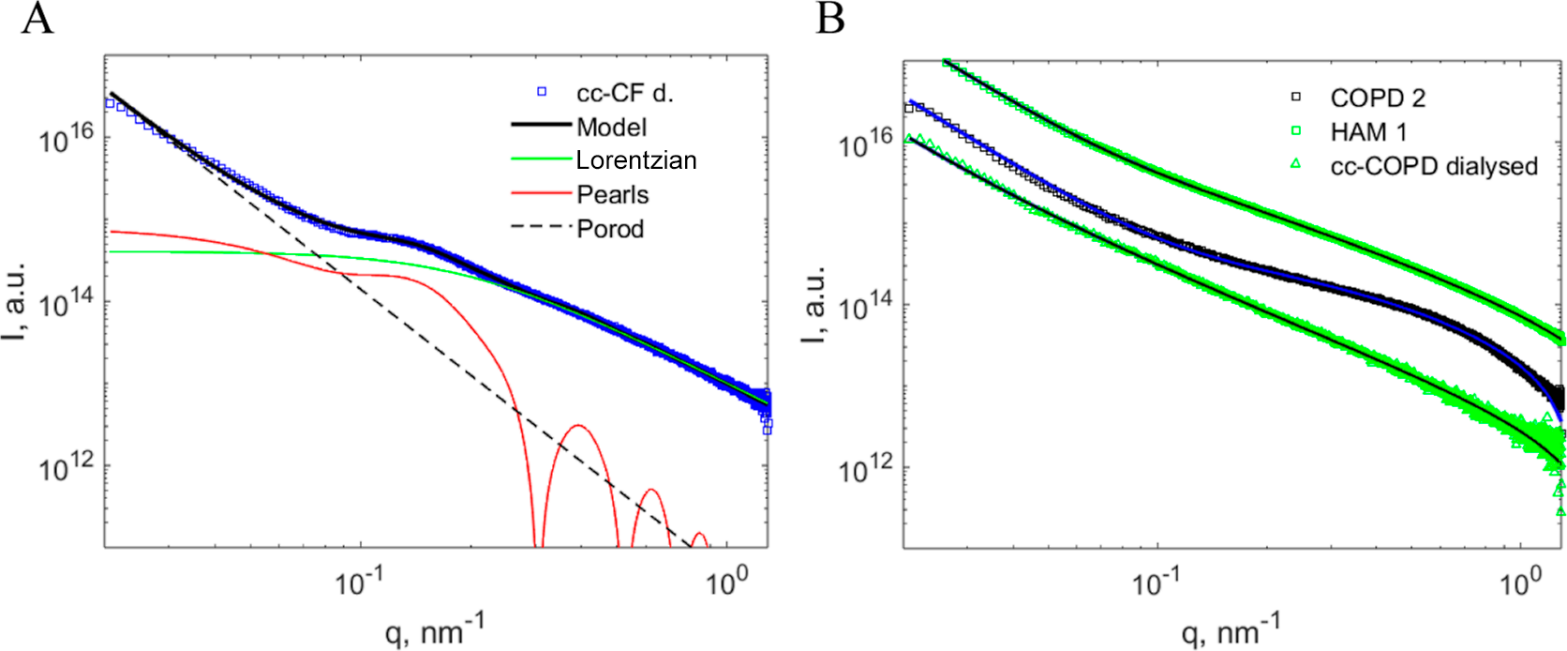
Modeling of scattering intensity for a dialyzed cc-CF sample (cc-CD d.). (A) The black line shows the total model fit, and other lines represent contributions from the three components of the model as specified in the legend. Parameters: *R* = 14.7 nm, *l* = 22.3 nm, ξ = 5.1 nm, *n* = 3.48, and *m* = 2.25. (B) Examples of modeling scattering intensity for 3 different samples using a correlation length model with fixed exponents *n* = 2.8 and *m* = 1.7.

All samples except dialyzed cc-CF showed the same shape of the scattering curves: a relatively straight line at low *q* values and a broad bump in the region 0.2–1.0 nm^–1^. These samples were modeled using the correlation length model (eq 8). The globule contribution was omitted because of the absence of any corresponding features on the scattering curves. We suggest that the reason for this behavior is a strong contribution from the mucin side chains that mask the scattering from globules. In contrast, in dialyzed cc-CF samples, a clear bump at 0.1–0.2 nm^–1^ was present, and the full model (eqs 7–9) was used to describe this data. An example of a fitting using this model is shown in Figure 8. The obtained globule radius is in good agreement with AFM data (Table 1), while the distance between the centers of globules (52 nm) is lower than measured by AFM, which can be attributed to a difference in conformations on the surface and in the bulk. The obtained correlation length ξ is rather short—around 5 nm, which agrees well with the AFM observation of shorter side chains in cc-CF.

As mentioned above, in the modeling of other samples, the contribution from the globules was not taken into account. Moreover, since some of the curves were relatively featureless, we chose to fix the values of Porod and Lorentzian exponents to values of 2.8 and 1.7, respectively. While the first parameter was chosen empirically based on the slopes of the intensity curves at low *q*, the second parameter not only correlates with the typical slopes at high *q* but also corresponds to the self-avoiding walk conformation of mucin side chains. Several examples of fitting using this approach are shown in Figure 8B, and a more complete set of data can be found in the Supporting Information (Table S2 and Figure S3). Having the constraints described above, the model is dependent on three fitting parameters: Porod scale, Lorentzian scale, and correlation length (ξ). While the scales are dependent on concentrations, the correlation length provides valuable information about the properties of molecules in different types of mucus.

**Figure 9:**
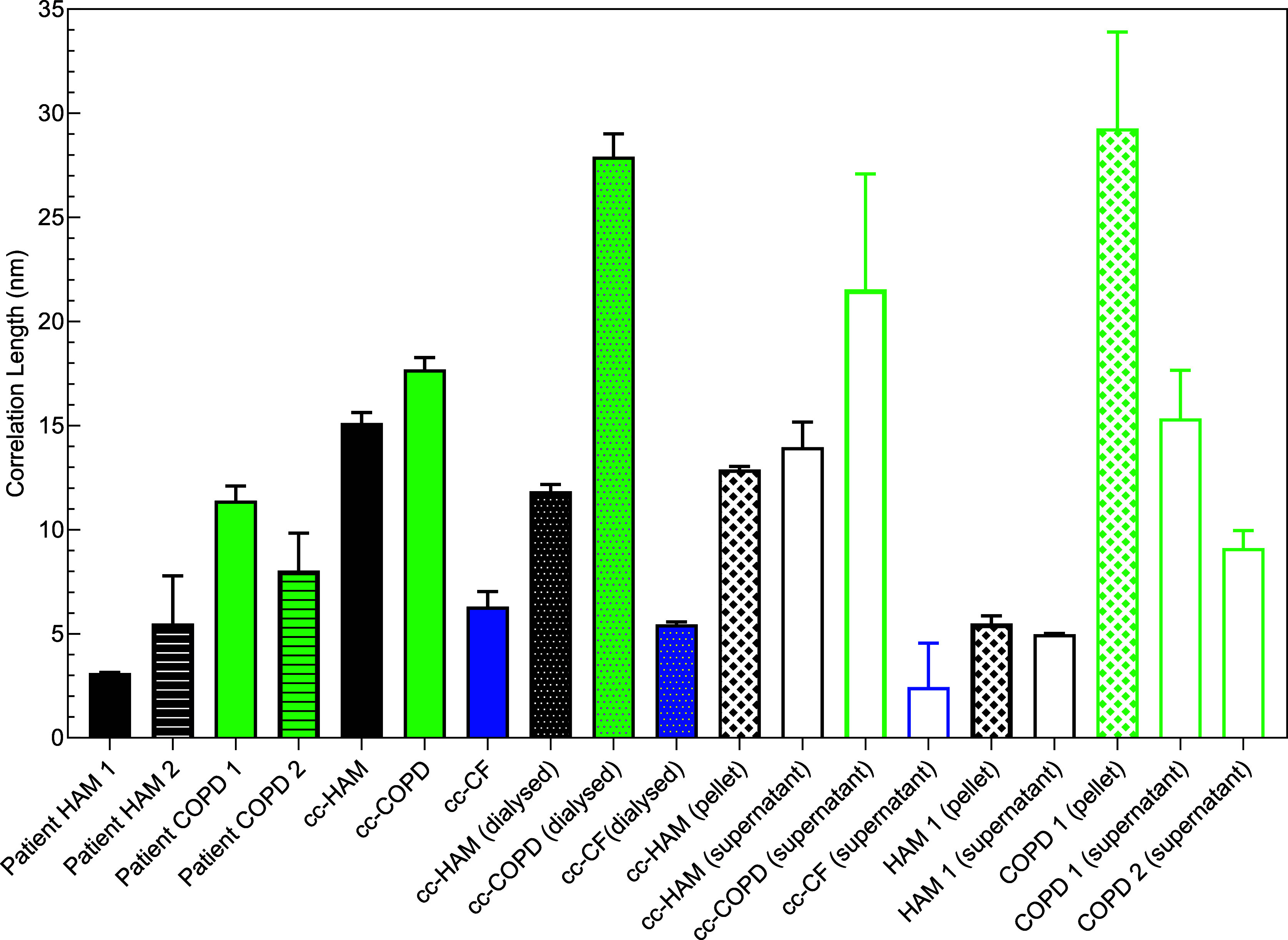
Correlation lengths determined by model fitting of the scattering curves from HAM and COPD patient mucus; cc HAM, COPD, and CF mucus; and the cc-HAM, cc-COPD, and cc-CF dialyzed samples. For all samples except cc-CF (dialyzed), the correlation length was determined using the correlation length model (eq 8), while for cc-CF (dialyzed) samples, the whole model that includes contributions from the globules (eqs 7–9) was used.

Figure 9 shows that within one type of sample, the correlation length is lowest for CF and highest for COPD samples. We suggest that this reflects the length of side chains: shorter chains correspond to lower correlation lengths. Indeed, assuming the same grafting density of the chains on the polypeptide backbone, longer chains would correspond to, on average, larger segment to segment distances between the side chains and also between the backbone and side chains.

### Hydration and Structure of Human Airway Mucus in Health and Muco-Obstructive Diseases

#### Structural Differences

Both the AFM and SAXS data presented above are consistent with the idea that mucin molecules can be presented as globules connected with glycosylated polymeric chains. The AFM data suggest that the globule sizes are increased in CF samples compared to HAM and COPD, while the polymeric spacers are shorter and thinner. To illustrate, we report a volume ratio (*V*_g_/*V*_S_, the ratio of the globule volume over the spacer volume) of a mucin monomer using the average features of the mucin molecules (Table 1), where a high volume ratio suggests the globules dominate the monomer volume. The cc-CF sample has a high *V*_g_/*V*_S_ ratio (3.3), which suggests the cc-CF mucin has a greater presence of the globules compared to the interlinking spacers. Similarly, SAXS data on CF samples feature a bump in the middle range of *q* values, absent in HAM and COPD samples, which is consistent with the AFM results, suggesting a more pronounced contribution from globules in CF samples.

Below, we propose an explanation for this observation based on the CF literature data. The condensed overall structure seen in the cc-CF sample could be explained by the mucin structure prior to secretion. During exocytosis, mucins are densely packed in concentrated and dehydrated granules, held together by calcium (Ca^2+^) ions that shield the repulsive forces of the negatively charged sialic acids in the mucins.^58^ Once secreted onto the extracellular surface and in the presence of HCO_3_^–^, the mucins unpack.^59,60^ However, in CF, the dysfunctional CF transmembrane conductance regulator channel reduces the amount of HCO_3_^–^ secreted into the ASL on the airway epithelium, resulting in incomplete unpacking and limited swelling of mucins.^61^ Since the glycosylated spacers in mucins are responsible for water uptake and the repulsion of nearby mucins, allowing mucins to disperse when in contact with water, we propose that the observed mucin organization found in the cc-CF mucus may indicate an incomplete unpacking and swelling of the backbone spacers. Combined with reduced side chain lengths, this leads to a reduction in the water-binding ability of these mucins (also seen as a low water content of the cc-CF mucus in the QCM-D analysis above).

#### Difference in Water Sorption Properties

The sorption isotherms reported above suggest the following sequence in the water sorption capacity of the studied types of mucins: CF, HAM, and COPD (in the ascending order of the absorbed amount of water), see Figure 2.

The different water activities seen in the sorption isotherms are thought to be influenced by different mucin types, their exocytosis, structure (in terms of carbohydrate vs protein content), and the presence of other molecules,^61^ which have been shown to cause variability in the mucus’s water content.^23^ The mucin’s glycosylated backbone is the structural feature responsible for the water content of mucus. Znamenskaya and co-workers^23^ suggested a high carbohydrate/protein ratio results in a greater water content at low relative humidity environments, while high concentrations of sialic acid regions, which impart greater electrostatic repulsive forces, resulting in a greater water content in high relative humidity environments.

According to our AFM and SAXS data, the globules in CF samples are increased, while hydrophilic spacers are shortened compared to those of other types of mucins. Since the globules are expected to be less hydrated than the unfolded glycosylated spacer, this explains the somewhat poorer water sorption properties of CF samples. The limited ability to absorb water seen in the CF mucin samples can be interpreted as an increase of mucin density.^62^

Contrary to the low sorption properties of the cc-CF, COPD 1, COPD 2, and cc-COPD mucus had the highest water sorption at each humidity point, suggesting that the COPD mucus has a higher affinity for water. A known characteristic of COPD mucus is a high gel-forming mucin concentration due to overproduction,^63−65^ particularly MUC5AC^65^ and MUC5B in an altered lower-charged glycosylated form.^66^ As mentioned above, structural factors, such as carbohydrate/protein ratios, affect the hydrophilicity of mucins. Therefore, the generally greater water sorption capacity of the COPD mucus in high relative humidity environments could be caused not only by mucin hypersecretion but also by the mucin structure itself. Mucins that have high carbohydrate/protein ratios and sialic acid contents, due to the higher hydrophilicity of carbohydrates and charged groups, exhibit a greater ability to absorb water.

In the experimental design for these sorption isotherms, the absorbed water was provided by the humidified air, and the water content is lower than reported in the literature, where the collected mucus is often diluted in the sputum.^67−69^ This may be caused by the water source in the experiments being restricted to air, whereas in vivo, this is not the only source of water for the mucus. Water in the mucus is also provided by the epithelial cells. In the case of COPD, where the overlaying mucus has a high mucin concentration and appears to be more hydrophilic than healthy or CF mucus with the higher water sorption values presented here, water is also absorbed from the periciliary layer, depleting the periciliary layer and swelling the overlaying mucus layer.^68^ This imbalance results in the ciliated epithelium being compressed and ineffective mucociliary clearance, which elicits cough. Hyper-concentrated mucus may have a protective role in preventing damage to the airway epithelium due to the evaporation of water from the airway surface liquid, which can be caused by increased ventilation or inappropriately humidified air during noninvasive and invasive respiratory support. However, when mucus is produced in excess, this leads to airway obstructions, which promote respiratory infections and affect the primary role of the lungs to provide efficient gas exchange. Airway hydration involving humidification of respiratory gases during ventilation or respiratory support^70^ and administration of nebulized saline^71^ are used to affect the mucus composition to improve mucociliary transport and clinical outcomes.^26,72^

The study’s results, though subject to inherent limitations, provide initial findings to begin understanding how water sorption properties and mucin structures change in muco-obstructive disease. Notably, the small sample size compromises the statistical power and generalizability, and the inherent natural variability between patient samples cautions against broad generalizations. Nevertheless, this study helps to establish an understanding of pathophysiological mechanisms in muco-obstructive diseases.

## Conclusions

The sorption properties and nanostructures of the mucins found in the available HAM, COPD, and CF mucus samples investigated in this study were significantly different. From the QCM-D sorption isotherms, there was a significant difference in the sorption properties of the HAM, COPD, and CF mucus, while the AFM and SAXS data provided insights into mucin structure that helped to explain this difference. Mucin structures differed by the hydrophilic spacer lengths, hydrophobic globule sizes, and types of chain arrangements. The different structures observed in the COPD and CF mucins suggest different abilities of water to associate with the mucins. In COPD mucins, the longer and more hydroxylated hydrophilic spacers correlate with higher water contents reported by QCM-D, while the shorter and less hydroxylated hydrophilic spacers in CF mucins correlate with lower water contents also found in QCM-D.

In addition to the water contents, the sorption isotherms of nondialyzed mucus revealed a lack of hydration-induced glass transition, which suggests mucus remains in a rubbery/liquid state. Mucus maintaining a rubber state in low relative humidity environments highlights its protective properties, which help to prevent desiccation of the airway surface. However, when mucus is dialyzed, thereby removing small molecules, a dehydration-induced glass transition becomes apparent. The small molecules found in mucus act as plasticizers, maintaining molecular mobility and allowing the mucus to remain rubbery.

The study demonstrates the variability of the physical properties of mucus and may indicate a potential difference of mucus in muco-obstructive lung disease that could play a pathophysiological role apart from the commonly observed hyperconcentration and increased volume of sputum. Further research is required to investigate the properties of mucus derived from larger patient populations. In addition, further investigations into how small molecules interact with mucins and how they contribute to mucus water sorption capacity would improve our understanding of the physiological mechanisms of mucociliary transport in muco-obstructive diseases.

## Supporting Information Available

Comparison test between water sorption properties of the different samples, AFM images of the samples, SAXS model fitting results, SAXS model fit curves, and SAXS curves from the samples (PDF)
