# Supplementary material for: Water Sorption and Structural Properties of Human Airway Mucus in Health and Muco-Obstructive Diseases
Source: Biomacromolecules. 2024 Feb 9;25(3):1578–91. doi: 10.1021/acs.biomac.3c01170 (PMC10934264; doi:10.1021/acs.biomac.3c01170)
Supplement: Supplementary file 1 — bm3c01170_si_001.pdf [file bm3c01170_si_001.pdf]

Supporting Info

Water sorption and structural properties of airway mucus in health and muco-obstructive diseases

*Susyn J. Kelly [a,b**], Vladislav Genevskiy [c,d**], Sebastian Björklund [c,d], Juan F. Gonzalez-Martinez [e], Lara Poeschke [f], Maik Schröder [f], Georg Nilius [g,f], Stanislav Tatkov [a], Vitaly Kocherbitov [c,d*]*

[a] Fisher & Paykel Healthcare Ltd., 15 Maurice Paykel Place, East Tamaki, Auckland, NZ-2013, New Zealand.

[b] Department of Clinical Sciences, Ross University of Veterinary Medicine, Basseterre, KN-0101 Saint Kitts and Nevis.

[c] Biomedical Science, Faculty of Health and Society, Malmö University, Malmö, SE-20506, Sweden.

[d] Biofilms Research Center for Biointerfaces, Faculty of Health and Society, Malmö University, Malmö, SE-20506, Sweden.

[e] Department of Applied Physics, Universidad Politécnica de Cartagena, 30202 Spain

[f] Evang. Kliniken Essen-Mitte GmbH, Essen, DE-45136 Germany.

[g] Universität Witten/Herdecke, Witten, DE-58455, Germany.

* Corresponding author

** These authors contributed equally to this work

Table S1: One-way ANOVA with post-hoc Tukey multiple comparison test for significant difference in maximum water contents when different mucus samples were exposed to 99% relative humidity. Ns = not significantly different, * is significantly different ($P < 0.05$), and ** is very significantly different ($P \ll 0.05$).

**Tukey's multiple comparisons test**	**Significant Difference**	**Adjusted P Value**
HAM vs. cc-HAM	ns	0.874
COPD 1 vs. cc-COPD	ns	0.580
COPD 2 vs. cc-COPD	ns	0.146
COPD 1 vs. COPD 2	ns	0.770
HAM vs. COPD 1	*	0.030
HAM vs. COPD 2	**	0.009
cc-HAM vs. cc-COPD	*	0.0473
cc-HAM vs. cc-CF	**	<0.009

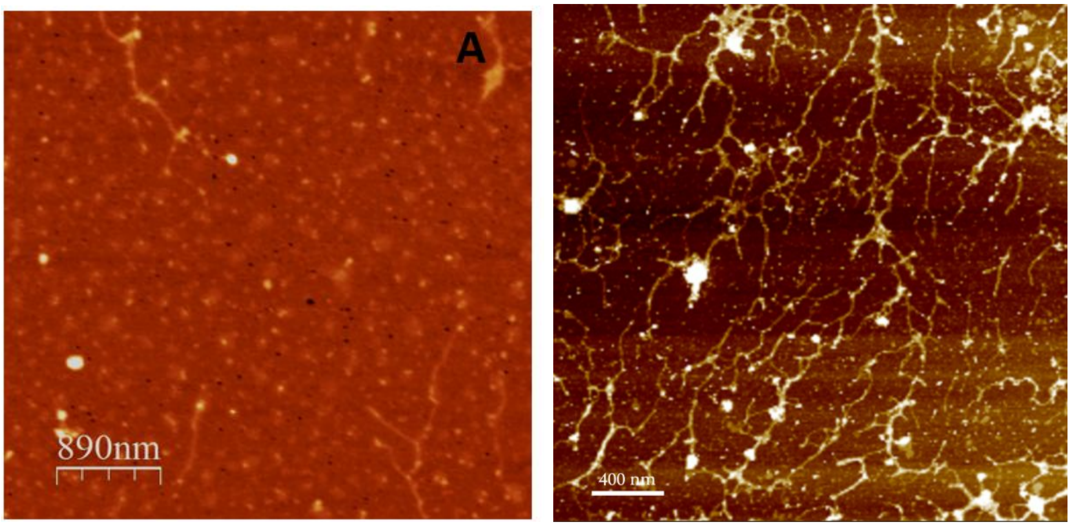

Figure S1. Atomic force microscopy of (A) healthy airway mucus (HAM) and (B) cell-culture chronic pulmonary disorder (cc-COPD) mucus showing large amounts of small molecules surrounding the fiber-like mucin molecules.

Table S2. Fitting results from the scattering curves of mucus samples using the correlation length model (fixing the Porod exponent to 2.8 and the Lorentz exponent to 1.7) and * linear pearl model. Model fits can be seen in Figure S3.

Samples	Lorentz Scale	Porod Scale	Correlation Length	Fit Quality	slope (-n)	Flory exponent (v)
Patient HAM 1	329	0.00098	31.21	4.669	2.6271	0.3806
Patient HAM 1	336	0.00088	31.44	5.2417	2.6601	0.3759
Patient HAM 1	333	0.00082	30.97	5.2832	2.654	0.3801
Patient HAM 2	502	0.01502	28.53	62.79	3.2056	0.3945
Patient HAM 2	2174	0.01596	70.78	46.828	3.5049	0.3874
Patient HAM 2	1784	0.00974	65.25	26.572	3.7789	0.3895
Patient COPD 1	5328	0.00790	113.08	8.4353	2.5809	0.3875
Patient COPD 1	5932	0.00794	121.31	8.029	2.5734	0.3886
Patient COPD 1	4818	0.00792	107.26	8.924	2.5812	0.3854
Patient COPD 2	4533	0.00660	102.63	2.3359	2.4921	0.4013
Patient COPD 2	3376	0.00552	80.71	1.609	2.4949	0.4008
Patient COPD 2	3158	0.00423	79.50	7.935	2.4891	0.4023
Patient COPD 2	1959	0.00608	58.39	9.003	2.4957	0.4011
ccHAM	1878	0.00163	152.8	1.67	2.8647	0.3491
ccHAM	1762	0.00171	145.85	2.088	2.8788	0.3474
ccHAM	1815	0.00164	148.77	1.958	2.8711	0.3458
ccHAM	1993	0.00158	157.48	2.185	2.7991	0.3487
ccCOPD	2147	0.00130	184.25	2.096	2.9369	0.3405
ccCOPD	1895	0.00140	170.85	1.924	2.923	0.3421
ccCOPD	1952	0.00130	178.32	1.833	2.9601	0.341
ccCOPD	1813	0.00134	174.78	1.859	2.9298	0.3441
ccCF	1919	0.00327	54.53	14.462	2.738	0.3652
ccCF	2000	0.00290	59.62	7.430	2.8515	0.3507
ccCF	2283	0.00274	70.13	9.109	2.7891	0.3589
ccCF	2338	0.00278	68.07	8.492	2.7963	0.3601
ccHAM (dialysed)	348	0.00054	117.48	0.94131	3.0174	0.3314
ccHAM (dialysed)	388	0.00053	122.71	0.92756	3.0195	0.3312
ccHAM (dialysed)	369	0.00056	118.69	1.0483	3.0152	0.333
ccHAM (dialysed)	351	0.00056	115.09	0.94138	3.0201	0.3296
ccCOPD (dialysed)	1322	0.00031	288.22	1.168	3.0574	0.3271
ccCOPD (dialysed)	1143	0.00039	263.36	1.12	3.0544	0.3274
ccCOPD (dialysed)	1328	0.00034	284.91	1.131	3.0568	0.3273
ccCOPD (dialysed)	1283	0.00034	280.22	1.145	3.0554	0.3256
ccCF(dialysed)*	443	4.20E-06	54.112	3.2613	3.0329	0.3297
ccCF(dialysed)*	454	4.27E-06	54.44	3.294	3.037	0.3293
ccCF(dialysed)*	437	3.69E-06	53.293	3.176	3.047	0.3289
ccCF(dialysed)*	475	2.92E-06	56.239	3.01	3.056	0.3288
ccHAM (pellet)	1441.5	0.0022629	128.31	1.8618		
ccHAM (pellet)	1481	0.00217	130.56	1.9061		
ccHAM (pellet)	1422	0.00211	127.61	1.717		
ccHAM (supernant)	1312	0.00146	125.7	1.9114		
ccHAM (supernant)	1729	0.00132	146.8	2.24		
ccHAM (supernant)	1647	0.00126	146.35	2.213		
ccCOPD (supernant)	1321	0.0014	157.2	2.3491		
ccCOPD (supernant)	2316	0.00165	221.6	1.926		
ccCOPD (supernant)	3276.4	0.00103	267.6	2.052		
ccCF (supernant)	1268	0.0052	35.401	47.19		
ccCF (supernant)	1369	0.0042	37.62	36.43		
HAM (pellet)	419	0.00461	56.606	1.7614		
HAM (pellet)	569.24	0.0040172	57.607	1.346		
HAM (pellet)	333.05	0.00413	50.946	1.985		
HAM (supernant)	312	0.000418	49.669	2.011		
HAM (supernant)	321.75	0.00047	49.521	1.862		
HAM (supernant)	327.88	0.00043	50.252	1.89		
COPD 1 (pellet)	6946.2	0.00342	306.44	2.0224		
COPD 1 (pellet)	4743.7	0.0034	241.1	1.479		
COPD 1 (pellet)	8409	0.0035	330.7	1.967		
COPD 1 (supernant)	2398	0.00328	179.59	1.1346		
COPD 1 (supernant)	1682.3	0.0031	144.74	1.066		
COPD 1 (supernant)	1523	0.0026	136.01	1.148		
COPD 2 (supernant)	410.12	0.000465	98.604	1.142		
COPD 2 (supernant)	386.6	0.000478	92.832	0.942		
COPD 2 (supernant)	320.48	0.00071	82.063	0.9347		

<table>
<tr><th></th><th>Patient Mucus</th><th>Cell-culture Mucus</th></tr>
<tr><td>HAM</td><td>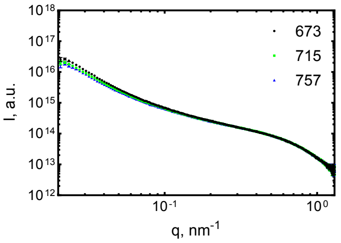

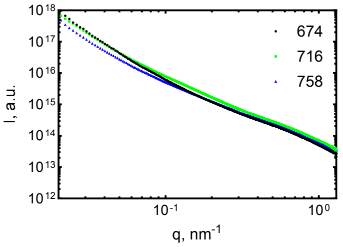
</td><td>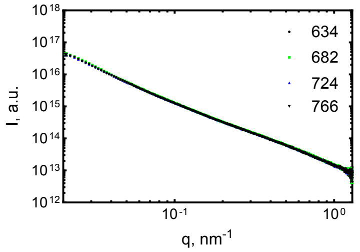
</td></tr>
<tr><td>COPD</td><td>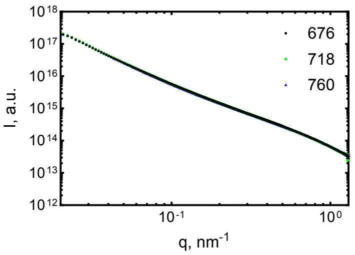

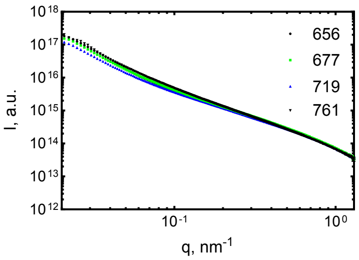
</td><td>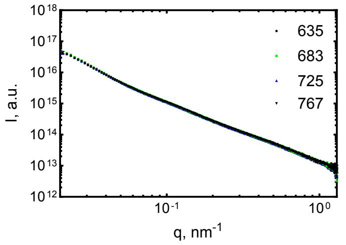
</td></tr>
<tr><td>CF</td><td></td><td>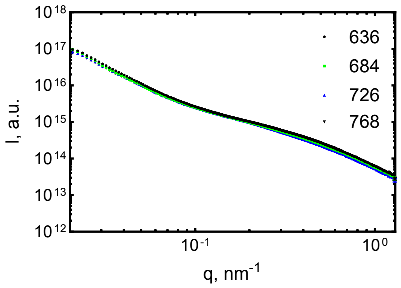
</td></tr>
</table>

Figure S2. All scatter curves from original mucus samples over the measured q-range (0.02 to 1.3 nm^{-1}) of patient mucus from healthy airway mucus (HAM) and chronic obstructive pulmonary disease (COPD), and cell-culture mucus from HAM , COPD and cystic fibrosis (CF).

	Patient Mucus	Cell-culture Mucus	Dialyzed Cell-culture Mucus
HAM	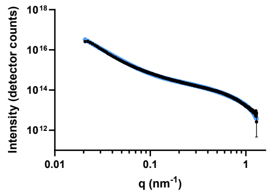	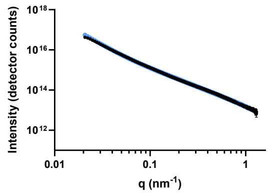	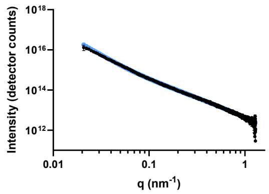
COPD	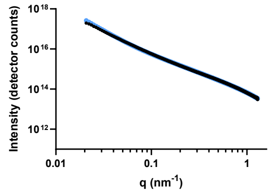	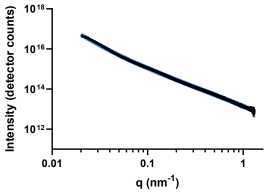	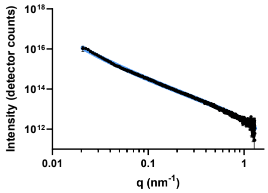
CF		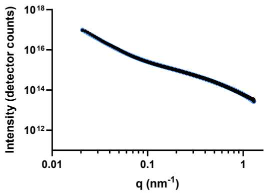	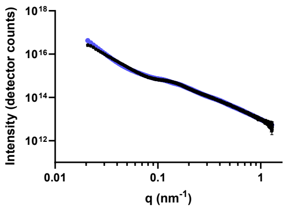

Figure S3. Representative correlation length model fitted (blue) and the correlation length with linear pearls model (purple) to the full q-range of scattering curves from healthy airway mucus (HAM 1), chronic obstructive pulmonary disease mucus (COPD 1) patient mucus; cell-culture (cc) HAM, COPD and cystic fibrosis (CF) mucus and the cc-HAM, cc-COPD and cc-CF dialyzed samples. Chi^2 values were (from top left to bottom right): 4.7, 1.7, 0.9, 8.4, 2.1, 1.2, 7.4, and 3.0).

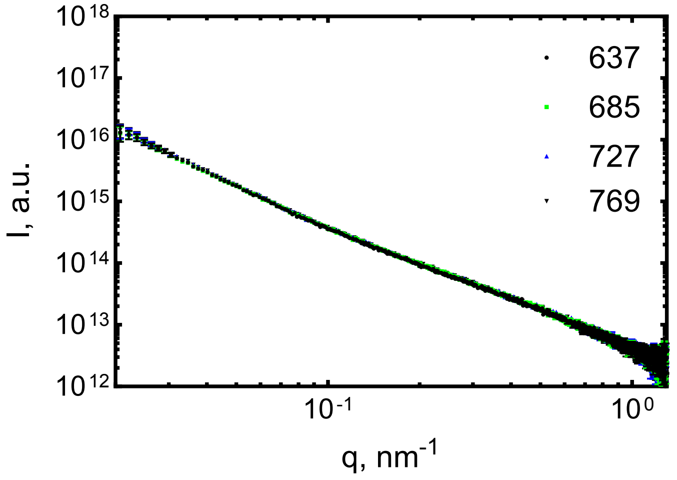
10^18
10^17
10^16
10^15
10^14
10^13
10^12
I, a.u.
10^-1
10^0
q, nm^-1
637
685
727
769

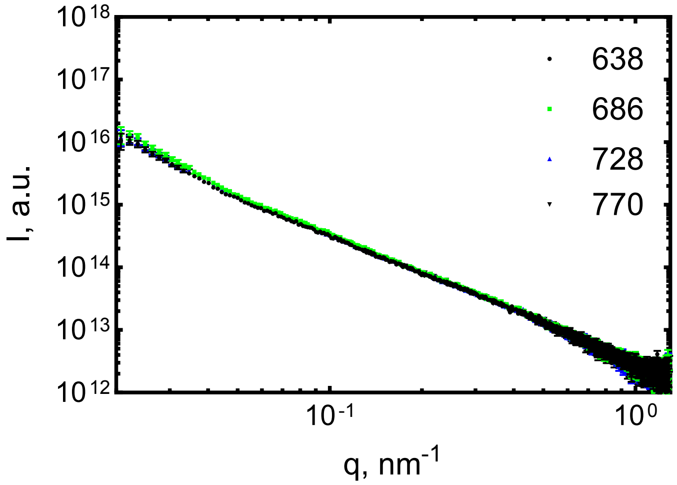
10^18
10^17
10^16
10^15
10^14
10^13
10^12
I, a.u.
10^-1
10^0
q, nm^-1
638
686
728
770

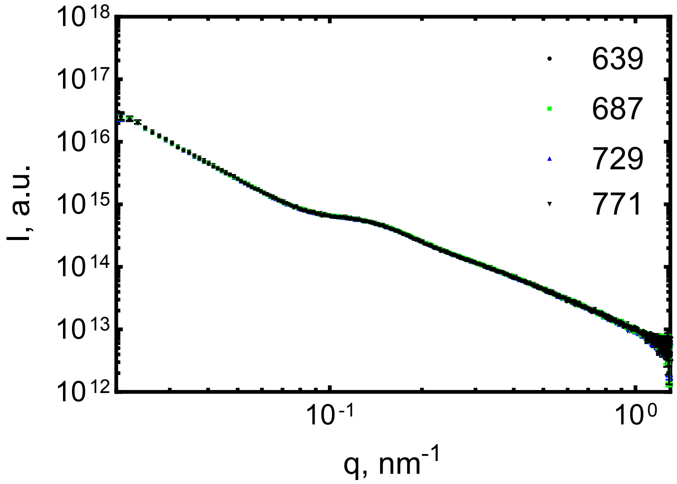
10^18
10^17
10^16
10^15
10^14
10^13
10^12
I, a.u.
10^-1
10^0
q, nm^-1
639
687
729
771

Figure S5. All scatter curves from dialyzed cc-HAM, cc-COPD and cc-CF samples.
